# Female Genital Tuberculosis

**DOI:** 10.1093/ofid/ofac543

**Published:** 2022-10-21

**Authors:** Christine Tzelios, Werner M Neuhausser, David Ryley, Nhi Vo, Rocio M Hurtado, Ruvandhi R Nathavitharana

**Affiliations:** Department of Medicine, Harvard Medical School, Boston, Massachusetts, USA; Division of Reproductive Endocrinology and Infertility, Beth Israel Deaconess Medical Center and Harvard Medical School, Boston, Massachusetts, USA; Division of Reproductive Endocrinology and Infertility, Beth Israel Deaconess Medical Center and Harvard Medical School, Boston, Massachusetts, USA; Boston IVF, Boston, Massachusetts, USA; Division of Radiology, Beth Israel Deaconess Medical Center, Boston, Massachusetts, USA; Division of Infectious Diseases, Massachusetts General Hospital and Harvard Medical School, Boston, Massachusetts, USA; Division of Infectious Diseases, Beth Israel Deaconess Medical Center and Harvard Medical School, Boston, Massachusetts, USA

**Keywords:** female genital TB, infertility, reproductive tract TB, tuberculosis

## Abstract

Female genital tuberculosis (FGTB) is an important cause of morbidity and infertility worldwide. *Mycobacterium tuberculosis* most commonly spreads to the genital tract from a focus elsewhere in the body and affects the bilateral fallopian tubes and/or endometrium. Many patients with FGTB have indolent disease and are only diagnosed after evaluation for infertility. Women may present with menstrual irregularities, lower abdominal or pelvic pain, or abnormal vaginal discharge. Given the low sensitivity of diagnostic tests, various composite reference standards are used to diagnose FGTB, including some combination of endoscopic findings, microbiological or molecular testing, and histopathological evidence in gynecological specimens. Early treatment with a standard regimen of a 2-month intensive phase with isoniazid, rifampin, ethambutol, and pyrazinamide, followed by a 4-month continuation phase with isoniazid and rifampin, is recommended to prevent irreversible organ damage. However, even with treatment, FGTB can lead to infertility or pregnancy-related complications, and stigma is pervasive.

A 33-year-old female scientist who moved to the United States (US) from China 1 year prior was referred to the infectious diseases clinic as part of an evaluation for infertility, given a prior history of tuberculosis (TB). She reported being diagnosed at age 24 with pulmonary, intestinal, and pelvic TB, for which she was treated for 1 year with combination antibiotic therapy, the details of which were unknown but included rifampin and isoniazid, with reported subsequent resolution of abnormal imaging findings, including fluid in the pelvis.

Computed tomography (CT) of the chest did not have parenchymal findings suggestive of active TB disease, but demonstrated calcified granulomas with some associated parenchymal scarring consistent with prior TB. Sputum cultures and nucleic acid amplification testing were negative. Hysterosalpingogram revealed that the right fallopian tube had a rigid pipe-stem appearance and the left fallopian tube had a nodular appearance with multiple strictures presumed to be from prior TB (Figure 1). Repeat endometrial specimens obtained from hysteroscopy and tissue obtained from laparoscopy, peritoneal biopsies, and bilateral salpingectomy were negative on mycobacterial smear and culture. TB polymerase chain reaction (PCR) was also negative. Pathology was not consistent with any features of acute infection, including TB, but demonstrated focal luminal obliteration of the fallopian tubes and transmural endometriosis, along with areas of fibrosis and focal chronic inflammation in the parametrium and pelvic side wall, and focal calcification in the perirectal tissue. These pathologic findings, coupled with her prior history of TB and calcifications on CT imaging, suggested that prior TB may have contributed to her infertility.

**Figure 1.**
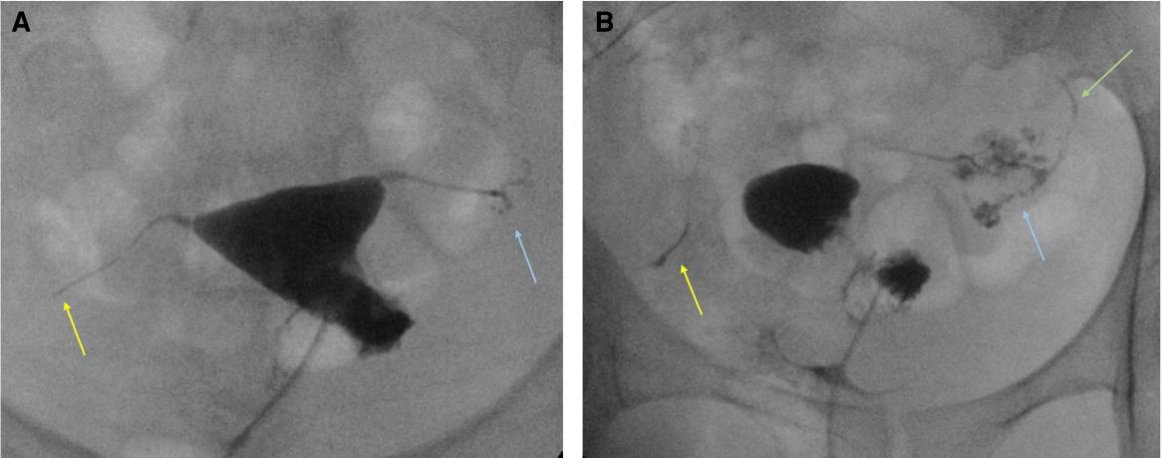
Images from fluoroscopic hysterosalpingogram. *A*, Anteroposterior fluoroscopic spot image of the pelvis demonstrates contrast opacification of a normal uterine cavity, but abnormal rigid pipe-stem appearance of the right fallopian tube with abrupt termination (left arrow) and no spillage of contrast into the peritoneum. The left fallopian tube similarly demonstrates a rigid appearance proximally. Distally, the left fallopian tube demonstrates multifocal strictures and beaded appearance (right arrow). *B*, Delayed oblique fluoroscopic spot image of the pelvis demonstrates persistent rigid pipe-stem appearance of the right fallopian tube (left arrow). Multifocal beaded and irregular appearance of the left fallopian tube is better appreciated on delayed imaging (middle arrow). There was abnormal, trace spillage of contrast into the peritoneum from the left fallopian tube (right arrow).

Here we present a narrative review to address common questions that arise during the clinical evaluation and management of people with female genital tuberculosis (FGTB). While practices may vary based on healthcare and country settings, including the availability of recommended diagnostic tests and imaging modalities, we sought to provide an evidence-based approach to guide clinicians irrespective of setting.

## EPIDEMIOLOGY

Tuberculosis affects an estimated 10 million people each year and is a leading cause of mortality globally [1]. Although *Mycobacterium tuberculosis* (*Mtb*) typically affects the lungs, it can also spread to other sites including the lymph nodes, pleura, bones, joints, meninges, and urogenital tract. Extrapulmonary tuberculosis (EPTB) represents 16% of reported cases globally, with significant regional variation [1].

FGTB is a rare yet important cause of morbidity and infertility in countries with a high overall prevalence of TB. The incidence of FGTB is challenging to estimate, as many people have asymptomatic disease and are only diagnosed after evaluation for infertility. Incidence of FGTB at infertility clinics varies geographically, from <1% in the US [2], to 2% in Italy [3], 4% in Saudi Arabia [4], 7% in Yemen [5], 17% in Nigeria [6], 2%–20% in Pakistan [7, 8], 6%–21% in South Africa [9, 10], and 3%–26% in India [11–13], likely due to differences in the overall prevalence of TB, clinical awareness of FGTB, and regional variation in the availability and use of sensitive and accurate diagnostic tests [14]. However, many estimates are from older studies with small sample sizes and, therefore, may not accurately represent the current epidemiological distribution.

FGTB commonly presents in women of reproductive age, occurring less frequently in postmenopausal women [14, 15]. The reasons for the higher prevalence of postmenopausal FGTB in high-income countries are not well understood, but differences in childbearing age that impact endometrial atrophy have been postulated to be implicated [14].

## PATHOGENESIS

*Mycobacterium tuberculosis* is transmitted from person to person through infectious droplets suspended in the air and inhaled into the lungs. FGTB almost always occurs secondary to a focus elsewhere in the body (typically, the lungs) and reaches the genital tract via hematogenous dissemination, lymphatic spread, or direct spread from adjacent abdominal organs [14, 16]. There is some evidence to suggest FGTB can be acquired as a primary infection via sexual transmission, although data are limited. A retrospective study of 128 women with FGTB found that 3%–5% of their male partners also had active genitourinary TB [17]. Furthermore, case studies have found the *Mtb* strains of sexual partners affected by genitourinary TB to be molecularly identical [18, 19]. There is no evidence of sexual transmission from a partner with pulmonary tuberculosis (PTB). FGTB may persist as a chronic infection, defined as the presence of *Mtb* in the genital tract, detected by PCR, without laparoscopic, microbiological, or histopathological evidence of disease, which may represent early subclinical infection that can later progress to active disease and impact ovarian reserve [20, 21].

The fallopian tubes are affected, typically bilaterally, in 90%–100% of cases of FGTB. Tubal involvement manifests as salpingitis with the formation of hydrosalpinx, pyosalpinx, tubo-ovarian masses, adhesions, and tubal obstruction [22]. Infection spreads from the fallopian tubes to the endometrium through direct drainage. The endometrium is affected in 50%–80% of cases, commonly manifesting as uterine cavity distortion and intrauterine adhesions [23, 24]. The ovaries are involved in 20%–30% of cases, with findings of adhesions, caseation, adnexal cysts, tubo-ovarian masses, and defective ovarian reserve [16]. Involvement of the cervix (5%–15% of cases), or the vagina or vulva (1%–2%) is more rare [16].

## CLINICAL PRESENTATION

Most patients with FGTB have indolent disease with no clinically significant symptoms and are often only diagnosed after evaluation for infertility. Infertility affects 60%–80% of women with FGTB and occurs due to distortion or obstruction of the fallopian tubes, intrauterine adhesions causing inadequate endometrial receptivity, or inflammatory destruction of ovarian tissue leading to defective ovarian reserve [14, 20, 25, 26]. Primary infertility (when pregnancy has never occurred) is more common than secondary infertility (when at least 1 prior pregnancy has occurred) and accounts for 66%–85% of infertility experienced by women with FGTB [8, 25, 27].

Following infertility, the most frequently reported symptoms in women of reproductive age are menstrual irregularities or other nonspecific symptoms such as lower abdominal or pelvic pain and abnormal vaginal discharge (Table 1) [14, 28]. Asherman syndrome (intrauterine adhesions alongside infertility and menstrual irregularities, occurring more commonly due to the sequelae of surgery or pelvic radiation) can also be caused by FGTB [29]. In postmenopausal women, FGTB is characterized by postmenopausal bleeding, leukorrhea, and pyometra [15]. Women of all ages may also experience weight loss and anorexia. Other constitutional symptoms, including malaise, night sweats, and fever, occur less commonly and may indicate concurrent infection elsewhere [21, 30].

**Table 1.** Clinical Presentation of Female Genital Tuberculosis

Symptoms			
Premenopausal	Postmenopausal	Clinical History	Examination Findings
Primary or secondary infertility Menstrual irregularities Hypomenorrhea Oligomenorrhea Menorrhagia Amenorrhea (primary or secondary) Dysmenorrhea	Postmenopausal bleeding Leukorrhea Pyometra	Travel or residence in a country with high tuberculosis prevalence Personal or family history of tuberculosis Primary or secondary infertility Recurrent implantation failure with in vitro fertilization	Abdominopelvic tenderness Adnexal or pelvic mass Ascites or abdominal distention Cervical or vulval growth Fever Pallor
All Ages			
Abdominal or pelvic pain Abnormal vaginal discharge Dyspareunia Postcoital bleeding Weight loss/anorexia Less common: constitutional symptoms (malaise, night sweats, cough, nausea, vomiting) and urinary symptoms			

The differential diagnosis for FGTB is broad. FGTB can mimic ovarian and endometrial carcinoma and may be detected incidentally during evaluation for malignancy [15, 31–33]. Geographic location, personal and family history of TB, and patient age can help guide diagnosis. FGTB is more common among women of reproductive age, whereas ovarian and endometrial cancers affect older women [32]. Elevated serum CA-125 levels often prompt suspicion of malignancy but may also be elevated in FGTB [32, 34]. Other biomarkers, like human epididymis protein 4, may be more specific for ovarian and endometrial cancer and, therefore, more useful when evaluating for malignancy among people being evaluated for TB [34]. Rarely, FGTB occurs concurrently with genital carcinoma [31, 35].

**Table 2.** Diagnostic Accuracy of Microbiological and Molecular Tests for Female Genital Tuberculosis

Test	Specimen	Sensitivity, %	Specificity, %	Reference Standard	References
AFB microscopy (ZN stain)	Endometrial or ovarian biopsy	1.4–8.4	100	CRS based on some combination of smear, culture, PCR, histopathology, laparoscopy, radiological findings, and response to ATT	Chopra et al [40]; Lu et al [41]; Sethi et al [42]
		21.8	100	Laparoscopy	Bhanothu et al [43]
AFB culture (solid media)	Endometrial or ovarian biopsy	8.6–41.8	100	CRS based on some combination of symptoms, smear, culture, PCR, histopathology, laparoscopy, radiological findings, and response to ATT	Chopra et al [40]; Lu et al [41]; Paine et al [44]
		42.1	99	Laparoscopy	Bhanothu et al [43]
	Menstrual blood	40.3	100	CRS based on histopathology and PCR	Paine et al [44]
AFB culture (liquid media)	Endometrial biopsy	7.1–40	90-100	CRS based on some combination of smear, culture, PCR, histopathology, and laparoscopy	Sethi et al [42]; Radhika et al [45]; Thangappah et al [46]
Xpert MTB/RIF	Endometrial biopsy/aspirates	11.1–50	100	CRS based on some combination of smear, culture, PCR histopathology, laparoscopy, and response to ATT	Sharma et al [47]; Sharma et al [48]; Tiwari et al [49]
		6.9–33	99.79–100	Culture	Sharma et al [47]; Ashwini et al [50]
TB-PCR	Endometrial biopsy	42.7–95.8	54–100	CRS based on some combination of symptoms, smear, culture, PCR, histopathology, laparoscopy, radiological findings, and response to ATT	Chopra et al [40]; Lu et al [41]; Sethi et al [42]; Paine et al [44]; Radhika et al [45]; Thangappah et al [46]; Tiwari et al [49]; Jindal et al [51]
		34.8	99.08	Culture	Ashwini et al [50]
		57.1–88.1	100	Laparoscopy	Bhanothu et al [43]; Bhanu et al [52]
	Menstrual blood	72.3–90.2	82.9–86.1	PCR (± histopathology)	Chaubey et al [38]; Paine et al [44]
	Pouch of Douglas fluid	23.5	100	CRS based on histopathology and PCR	Bhanu et al [52]
TB-LAMP	Endometrial biopsy	66.2	92.67	CRS based on smear, culture, PCR, and histopathology	Sethi et al [42]
Histopathology	Endometrial biopsy	8.8–64.8	93.23–100	CRS based on some combination of smear, culture, PCR, histopathology, laparoscopy, radiological findings, and response to ATT	Chopra et al [40]; Lu et al [41]; Sethi et al [42]; Paine et al [44]; Thangappah et al [46]; Tiwari et al [49]
	Endometrial biopsy, ovarian biopsy, and pelvic aspirated fluids	51.5	99	Laparoscopy	Bhanothu et al [43]

Abbreviations: AFB, acid-fast bacilli; ATT, antituberculosis therapy; CRS, composite reference standard; LAMP, l oop-mediated isothermal amplification; PCR, polymerase chain reaction; TB, tuberculosis; ZN, Ziehl-Neelsen.

FGTB is also commonly misdiagnosed as chronic pelvic inflammatory disease (PID). Both conditions may present with elevated erythrocyte sedimentation rate, a nonspecific biomarker of inflammation. The lack of clinical response to broad-spectrum antibiotics for PID in a patient at epidemiologic risk should raise concern for TB [16].

## DIAGNOSTIC APPROACH

### Sampling

To diagnose FGTB, it is critical to obtain samples from the involved portion of the genital tract for microbiologic and histopathologic examination [36]. Specimens are commonly obtained by endometrial biopsy or aspirate or by cervical biopsy, which poses a major limitation since the endometrium and cervix are not always involved [14, 36, 37]. Moreover, endometrial biopsies are most useful if obtained postovulation during the late secretory phase. Dilation and curettage and laparoscopy facilitate specimen collection from less accessible sites; however, these procedures are invasive and contraindicated in women with PID, vaginismus, cervical stenosis, or coagulopathy, as they may induce acute flare-ups and long-term complications [38]. Testing menstrual discharge (obtained on day 2 of the menstrual cycle by instilling and aspirating 10–20 cc of normal saline in the vagina and/or sampling from the cervical milieu using a speculum) may also improve diagnostic yield [37, 39].

**Figure 2.**
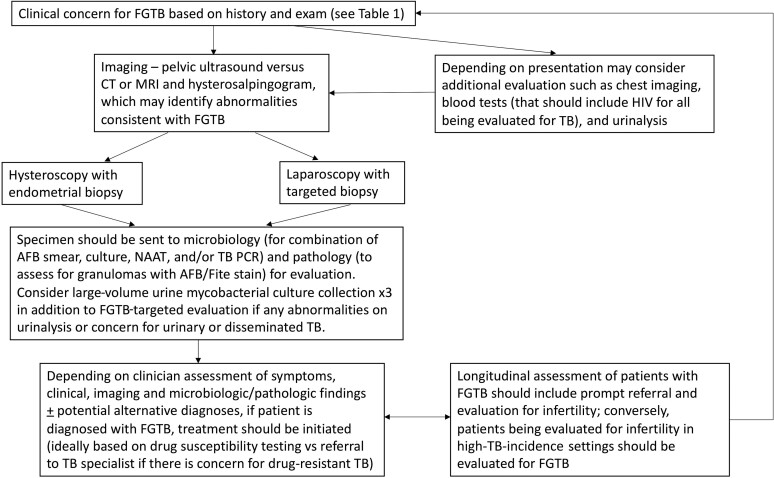
Suggested approach for the evaluation and management of people who may have female genital tuberculosis. Abbreviations: AFB, acid-fast bacilli; CT, computed tomography; FGTB, female genital tuberculosis; HIV, human immunodeficiency virus; MRI, magnetic resonance imaging; NAAT, nucleic acid amplification test; PCR, polymerase chain reaction; TB, tuberculosis.

### Microbiological Tests

Although acid-fast bacilli (AFB) microscopy remains widely used for the diagnosis of all forms of TB due to its relative rapidity and low cost, its sensitivity is low (1%–22%; Table 2) [40–43]. It requires at least 10^4^–10^6^ organisms/mL, which limits its utility since FGTB is paucibacillary [16]. AFB culture remains the reference standard for diagnosis and enables drug sensitivity testing, but results may take up to 8 weeks [36]. Compared to microscopy, culture has a slightly higher sensitivity, estimated between 7% and 42%, as only 10–100 bacilli/mL are required for detection; specificity is nearly 100% [16, 40–46, 49].

**Table 3.** Characteristic Findings of Female Genital Tuberculosis on Histopathologic Examination and Imaging

Test	Organ	Findings
Histopathology^a^	Endometrium, fallopian tubes, ovaries, and cervix	Small- to medium-sized, isolated, epithelioid granulomas, with or without caseation
Hysterosalpingography	Endometrium	Acute: venous and lymphatic intravasation of contrast due to destruction of the endometrium Chronic: filling defects, reduced uterine contractility, and distortion (T-shaped or small/shriveled) or complete obliteration of the uterine cavity caused by intrauterine adhesions from fibrosis and calcification
	Fallopian tubes	Beaded appearance caused by multiple constrictions along the tubes, or rigid pipe-like appearance from encasement of the tubes in dense, fibrous connective tissue Tubal dilation and filling defects caused by obstructive caseous ulcerations, fibrosis, or calcification at the junction between the tubal isthmus and ampulla
	Cervix	Stenosed cervical canal and a ragged, irregular contour with outpouchings caused by caseous ulcerations along the mucosa
Ultrasound	Endometrium	Thickened and distorted endometrium with heterogenous areas of hypoechoic foci caused by calcification or fibrosis
	Fallopian tubes	Edematous dilation and thickening of the fallopian tubes
	Ovaries	Hypoechoic foci of calcified granulomas, tubo-ovarian masses
Laparoscopy	Fallopian tubes	Tubercles, caseous nodules, beaded or pipe-like appearance of the fallopian tubes, tubo-ovarian masses
	Ovaries	Tubercles, caseous nodules, tubo-ovarian masses
	Pelvic peritoneum	Tubercles, caseous nodules, pelvic and perihepatic adhesions. In severe cases, extensive internal adhesions cause genital organs to become attached (frozen pelvis)
Hysteroscopy	Endometrium	Tubercles, caseous nodules, intrauterine adhesions, distorted or shrunken endometrial cavity

a While histopathology should be procured whenever possible, culture is more sensitive and should always accompany it.

### Molecular Tests

Nucleic acid amplification tests (NAATs) utilize PCR for more rapid, sensitive, and specific diagnosis (Table 2). The cartridge-based NAAT Xpert MTB/RIF assay (Xpert) is currently the only molecular test recommended by the World Health Organization (WHO) for detection of *Mtb* and rifampicin (RIF) resistance in EPTB [1].

For detecting EPTB, Xpert has good sensitivity (>80%) in urine and bone or joint fluid, and high specificity (>98%) in urine, cerebrospinal fluid, pleural fluid, and peritoneal fluid, when compared to culture [53]. However, studies evaluating the performance of Xpert in gynecological specimens are limited. Xpert has variable sensitivity, ranging from 7% to 50% on endometrial biopsy specimens, with higher sensitivity when compared to a composite reference standard rather than culture [47–50]. The specificity of Xpert is nearly 100%. Xpert Ultra and other molecular assays such as Truenat MTB-RIF have yet to be evaluated for FGTB.

Other real-time PCR assays that are not US Food and Drug Administration (FDA) approved or WHO recommended target a variety of *Mtb* genes, including IS6110, hsp65, and TRC4, and have reported sensitivities of 35%–96% and specificities of 54%–100% in endometrial biopsies [40–46, 49–52]. However, test performance may vary across laboratories, and concern for false positives and inability to detect RIF resistance render these tests less useful in clinical practice [16, 22].

Loop-mediated isothermal amplification (LAMP) is another WHO-approved NAAT for the diagnosis of TB, although it does not evaluate RIF resistance. It does not require sophisticated equipment and yields results in less than an hour. In a study of 300 patients in India, LAMP performed on endometrial biopsy specimens had a sensitivity of 66% and specificity of 93% [42].

### Biomarker Tests

The lateral flow lipoarabinomannan (LAM) assay is a rapid, point-of-care test that detects mycobacterial LAM antigen in urine and is currently the only biomarker-based assay recommended by the WHO for assisting in the diagnosis of PTB and EPTB in people living with HIV [54]. The test has yet to be evaluated specifically for FGTB.

### Tuberculin Skin Test and Interferon-γ Release Assay

Neither tuberculin skin test (TST) nor interferon-γ release assay (IGRA) is recommended for the diagnosis of active TB, and data for FGTB are limited. TST had a sensitivity of 55% and specificity of 80% compared to positive laparoscopic findings for FGTB [55]. The sensitivity and specificity of T-SPOT.TB (IGRA) in peripheral blood from women with confirmed FGTB were 86%–94% and 70%–75%, respectively, compared to a composite reference standard [41, 56]. Given these limited data suggesting that sensitivity may be low, a negative TST and/or IGRA cannot exclude potential FGTB.

### Histopathological Examination

Histopathological evidence of granulomas surrounded by giant epithelioid cells in genital tract specimens is often considered diagnostic for FGTB (Table 3) [36]; however, since culture remains most sensitive, attempts should be made to obtain a microbiological diagnosis [57]. Caseating endometrial granulomas are seen more frequently in postmenopausal women compared to women of reproductive age in whom the endometrial lining is often shed before caseation occurs [58]. Endometrial biopsies should preferably be obtained during the premenstrual or late secretory phase, as granulomas become more numerous and well-developed throughout the cycle [16, 58]. Estimates of histopathological examination (HPE) sensitivity for FGTB vary significantly from 9% to 65%, while specificity are commonly much higher [40–44, 46, 49]. Additional etiologies for uterine granulomas to consider include other infections, autoimmune diseases including sarcoidosis, systemic vasculitides, and prior surgery with subsequent foreign body reaction [59, 60].

### Radiologic Imaging

Hysterosalpingography is used to evaluate the uterine endometrium, tubal lumen, and cervix (Table 3). In the endometrium, findings of FGTB include intrauterine adhesions and a distorted or obliterated uterine cavity [24, 61]. The fallopian tubes may have a beaded appearance caused by multiple constrictions or a rigid pipe-like appearance in which the entire tube becomes encased in dense, fibrous connective tissue [61, 62]. Tubal obstructions, caused by granulomas, fibrotic tissue, or calcification, commonly occur at the junction between the tubal isthmus and ampulla [62]. The adnexa may show calcified lymph nodes or nodules [24]. Cervical involvement appears as a stenosed cervical canal with a ragged, irregular contour and/or diverticular outpouchings [24, 61].

Ultrasonography is often used to evaluate for uterine, tubal, and ovarian involvement. In FGTB, the endometrium appears thickened and distorted, and the endometrium and ovaries may contain hyperechoic foci of calcification or fibrosis [61]. The fallopian tubes, if involved, appear dilated and thickened [61].

Magnetic resonance imaging, CT scan, and positron emission tomography scan are helpful for detecting tubo-ovarian masses [36]. Chest radiograph (CXR) is used to identify concurrent or past PTB infection [36]. Abnormal CXR findings (healed TB lesions, hilar lymphadenopathy, fibrosis, opacities, and effusions) that may be consistent with TB are found in 8%–27% of women with FGTB [63–65]. One study of 37 men and women with genitourinary TB in the United Kingdom found that 13.5% had concurrent PTB [66].

### Endoscopic Procedures

Both laparoscopy and hysteroscopy reliably facilitate pelvic organ visualization and specimen collection; however, they are invasive and pose potentially significant surgical risks of excessive bleeding and infection flare-ups [14, 67, 68]. Endoscopic evidence of tubercles or caseous nodules is often considered diagnostic (Table 3). Other findings consistent with FGTB include pelvic and intrauterine adhesions, beaded or lead-pipe appearance of the fallopian tubes, hydrosalpinx, tubo-ovarian masses, and a distorted or shrunken endometrial cavity [69, 70]. In severe cases, extensive internal adhesions cause genital organs to become attached (“frozen pelvis”) [27].

### Composite Reference Standard

Given the low sensitivity of many diagnostic tests and the challenges of obtaining specimens, a composite reference standard (CRS) is often, if inconsistently, used (Figure 2). While the WHO consolidated guidelines for diagnosis discuss the accuracy of tests such as NAAT and LAM that can be used for the diagnosis of EPTB, there are no consensus guidelines for EPTB, including FGTB, that include recommendations for definition of a CRS. The Index TB guidelines for EPTB, developed by the WHO Country Office for India, suggest that confirmation of FGTB requires either laparoscopic findings, positive AFB smear or culture, or histopathological evidence of granulomas in a gynecological specimen [68]. Due to variable sensitivity and specificity of non-FDA-approved or non-WHO-recommended PCR tests, the Indian EPTB guidelines do not recommend treatment on the basis of these PCR tests alone, but decisions will vary in different clinical contexts such as a positive molecular WHO-recommended rapid diagnostic test (most commonly Xpert) that would typically lead to treatment being offered in a high-income country such as the US. Regardless of clinical setting, given the rising rates of drug resistance, attempting to obtain microbiologic confirmation is critical, as it enables drug susceptibility testing [71].

## TREATMENT

No studies have formally evaluated the optimal treatment regimen for FGTB. However, the WHO and Centers for Disease Control and Prevention/Infectious Diseases Society of America guidelines recommend that drug-sensitive EPTB be treated in the same manner as PTB for 6 months [72]. The standard regimen, which consists of a 2-month intensive phase with isoniazid, rifampin, ethambutol, and pyrazinamide followed by a 4-month continuation phase with isoniazid and rifampin has been used to treat FGTB, with low rates of disease recurrence [16, 37, 73]. We note that WHO guidelines conditionally recommend that in populations with known or suspected high levels of isoniazid resistance, new TB patients may receive isoniazid, rifampin, and ethambutol, as therapy in the continuation phase. With respect to duration, a randomized controlled trial of 175 women in India demonstrated no significant difference between 6-month and 9-month regimens for FGTB in terms of cure and recurrence rates [74]. Multidrug-resistant FGTB is treated with second-line drugs for 24 months, with a 6-month intensive phase and an 18-month continuation phase [75].

Early treatment can prevent permanent damage to pelvic organs [27]. There is a dearth of evidence to guide specific recommendations regarding treatment monitoring for FGTB, similar to other forms of EPTB. Treatment response is often based on longitudinal clinical and radiographic reassessment. Ideally, repeat sampling would be performed to evaluate for culture conversion (since resolution of HPE changes may lag and molecular testing may yield detectable DNA that may persist beyond culture conversion), but this is often precluded by the need for invasive tissue biopsy. In the absence of guidance, similar to PTB, we recommend interval assessment of clinical and radiographic evolution at 2 and 6 months. Failure to respond to treatment would be defined as clinical deterioration or worsening imaging findings, and should prompt resampling to enable microbiological reassessment given the concern for the presence or amplification of drug resistance or co-pathologies such as superinfection. Surgery may be performed, in conjunction with antibiotic treatment, to drain abscesses [14, 16, 67]. A comparison of laparoscopic findings before and after treatment in 50 women at a tertiary referral center in India found a significant decrease in tubercles, caseous nodules, encysted ascites, pyosalpinx, and beaded tubes; no change was observed in rates of pelvic and perihepatic adhesions or hydrosalpinx [27]. In other studies, ultrasound showed increased endometrial thickness following treatment, and hysteroscopy demonstrated decreased distortion of the uterine cavity and reduced prevalence of early-stage adhesions, while more advanced adhesions persisted [76, 77]. While data on sexual transmission and the duration of potential infectiousness for FGTB after treatment initiation are limited, it may be advisable to recommend patients use barrier protection during sexual activities for up to a month. If there are concerns for drug resistance, expert input is advised to ensure the adequacy of the treatment regimen prior to determining when to resume sexual activity without barrier protection.

## LONG-TERM SEQUELAE

FGTB often causes irreversible damage to genital organs, and women with FGTB have a poor prognosis for fertility, even after treatment. Estimates of posttreatment conception rate vary from 12% to 23% [5, 11, 16, 78]. Although therapy is not always recommended on the basis of PCR alone, there is some evidence that a positive PCR in the absence of clinical findings may indicate subclinical disease and that early therapy can prevent extensive damage to the genital organs, thereby averting permanent infertility [22, 79].

For women who are unable to conceive spontaneously following treatment, other options are available. Surgery to correct anatomic abnormalities and ovulation induction yield low pregnancy rates, even after antibiotic treatment. In vitro fertilization (IVF) is the most successful infertility treatment and is recommended in cases where the fallopian tubes are damaged but the endometrium remains intact or minimally scarred [80, 81]. However, even after successful treatment for FGTB, the prognosis for infertile patients undergoing IVF remains poor. Parikh et al reported a 19.2% conception rate, 16.6% pregnancy rate, and 7.2% live birth rate [82]. The high rate of pregnancy loss in these patients likely indicates residual structural damage to the uterine cavity [82]. Of note, undergoing IVF prior to FGTB treatment can pose life-threatening complications to the mother and child, including risk of congenital TB and reactivation of pelvic TB, thus underscoring the importance of early evaluation and treatment for FGTB in women with infertility [83–87]. Bilateral salpingectomies are often performed prior to IVF to improve the odds of pregnancy and live birth in women with tubal disease, although it has not been studied in FGTB patients [88, 89]. In cases where the endometrium is destroyed but the ovaries remain undamaged, gestational surrogacy may be advised [22].

Following conception, former FGTB patients may be at risk for pregnancy-related complications [11, 22]. One study of 97 patients in India reported a posttreatment conception rate of 19% but a live birth rate of only 7%, attributed to ectopic pregnancies and spontaneous abortions [11]. Stigma also remains a pervasive burden faced by women with TB, which is exacerbated by the dual stigma created by infertility [90]. Box 1 provides a patient advocate perspective on this dual stigma that highlights the need for better knowledge of FGTB and patient-centered approaches to care delivery with insights for clinicians, policymakers, and affected communities.

Box 1. **Patient perspective provided from Gerry Elsdon, National Tuberculosis Ambassador, South Africa (text provided with permission, personal communication; see www.gerryrantselielsdon.co.za)**

One of the most daunting experiences for many women is learning that they might be unable to conceive a child. I was certain I could find a solution and underwent various treatments for infertility but to no avail. I eventually met a new fertility specialist who recommended a repeat biopsy of the uterus, specifically to look for endometrial tuberculosis (TB). Although I was now a television anchor travelling the world, I had grown up in a poor community in the Western Cape of South Africa where TB is highly prevalent. Yet when the results came back positive, I was shocked: *Had TB not been eradicated ages ago? I was afraid. How long have I had it? Was I infectious? Would I survive?* I realized that TB attacks indiscriminately and knows no borders.

Unlike many, I had access to the resources available to get me through the process but my private doctors were baffled by this diagnosis. To my surprise, I realised the quality of TB care would be higher in the government public health system rather than private health clinics. Yet it took time to find a clinic and I saw that the public system was overwhelmed by the number of people needing TB care and that people with TB were overwhelmed by the pain of stigma.

I underwent 9 months of treatment and it wasn’t easy. In fact after a month, given my lack of symptoms I wondered why I needed treatment but I stuck with it and at the end of treatment my biopsy results were negative for TB. However we subsequently discovered that I would not be able to carry a child, which carries dual stigma. Difficult as this was, I decided to use the media to tell my story of TB and infertility and to share stories of other people living with TB. I was appointed as a South African National TB Ambassador, Global TB Ambassador for the Federation of the Red Cross, and a TB Champion for the WHO StopTB Campaign, and work to increase knowledge about TB and fight the stigma surrounding it, so that others can be diagnosed and treated sooner.

## CONCLUSIONS

Despite having received what appears to be an adequate course of TB treatment, it is likely that our patient's prior pelvic TB left her with scarring that has impacted her fertility. She has now begun a course of IVF and hopes to conceive.

Although FGTB remains an uncommon extrapulmonary manifestation of TB, it should remain an important consideration for the evaluation of women presenting with pelvic symptoms, including infertility, in high-TB-incidence settings or those with epidemiological risk for TB (people who have lived in high-incidence settings, or who have had a prior diagnosis of TB or contact with someone who has had TB, or with abnormal imaging findings that could be suggestive of TB) who are in low-incidence settings (Figure 2). Targeted imaging, including hysterosalpingography, can help to identify findings such as tubal occlusion, beading, or calcifications, that may suggest active or prior TB. Obtaining specimens for microbiological diagnosis, ideally with mycobacterial culture that also enables drug susceptibility testing, should be a priority. Current treatment recommendations for 6 months are based on general guidance for EPTB. While data to guide treatment monitoring are limited, longitudinal clinical and imaging reassessment can help to identify patients whose treatment may not be effective to guide the need for repeated sampling. More accurate epidemiologic data for FGTB and standardized approaches to facilitate early diagnosis followed by prompt treatment are needed to avert some of its devastating long-term sequelae that often include infertility.

## Notes

***Author contributions.*** C. T.: Conceptualization, data curation, writing–original draft, review and editing. R. R. N.: Conceptualization, project administration, supervision, writing–original draft, review and editing. W. M. N., D. R., N. V., and R. M. H.: Writing–review and editing.

***Patient consent statement*.** The patient's written consent was obtained for publication. Ethics approval is not required otherwise for the publication of a case report with nonidentifying data and review of the literature.

***Financial support*.** C. T. is supported by an Infectious Diseases Society of America GERM Scholar Award. R. R. N. is supported by a National Institutes of Health Career Development Award (award number K23 AI132648-04) and an American Society of Tropical Medicine and Hygiene Burroughs Wellcome Fellowship.
